# Pituitary-adrenal function in patients with acute subarachnoid haemorrhage: a prospective cohort study

**DOI:** 10.1186/cc7084

**Published:** 2008-10-13

**Authors:** Stepani Bendel, Timo Koivisto, Esko Ruokonen, Jaakko Rinne, Jarkko Romppanen, Ilkka Vauhkonen, Vesa Kiviniemi, Ari Uusaro

**Affiliations:** 1 Department of Intensive Care, Kuopio University Hospital and Kuopio University, Puijonlaaksontie 2, 70211 Kuopio, Finland; 2 Department of Neurosurgery, Kuopio University Hospital, Puijonlaaksontie 2, 70211 Kuopio, Finland; 3 Eastern Finland Laboratory Centre, Kuopio University Hospital, Puijonlaaksontie 2, 70211 Kuopio, Finland; 4 Department of Medicine, Kuopio University Hospital and Kuopio University, Puijonlaaksontie 2, 70211 Kuopio, Finland; 5 IT Centre, Kuopio University PO Box 1627, 70211, Finland

## Abstract

**Introduction:**

Subarachnoid haemorrhage (SAH) may damage the hypothalamo-pituitary-adrenal gland (HPA) axis and disturb cortisol metabolism. There are no available data that relates to the response of the HPA axis in the acute phase of SAH. We aimed to characterise the behavior of serum adrenocorticotropic hormone (ACTH), total cortisol, stimulated total cortisol and free cortisol concentrations in acute aneurysmal SAH.

**Methods:**

A prospective cohort study was conducted of patients with acute aneurysmal SAH (n = 30) admitted to a tertiary university hospital. Patients admitted for elective aneurysmal surgery (n = 16) served as the control group. An ACTH stimulation test was performed twice during the first week and at three months. The main outcome measure was description of the ACTH-cortisol response by calculating serum free cortisol and measuring total cortisol and ACTH concentrations. A mixed models method was used for testing between the groups, allowing heterogeneity between the groups.

**Results:**

Patients with SAH had higher initial serum total cortisol (mean +/- SD; 793 +/- 312 nmol/L) and free cortisol concentrations (83 +/- 55 nmol/L) than control patients (535 +/- 193 nmol/L, p = 0.001 and 33 +/- 18 nmol/L, p < 0.001, respectively). Thereafter, there were no differences in this respect. Serum free and total cortisol concentrations correlated but were unaffected by the severity of SAH. ACTH concentrations were comparable between SAH and control groups. Patients with Hunt-Hess grades IV to V had higher ACTH concentrations at day one (10.7 +/- 7.1 pmol/l/L) and day five (8.2 +/- 7.7 pmol/L) than patients with grade I-III (day one: 3.8 +/- 2.0 pmol/L, p = 0.002; day five: 4.7 +/- 1.8 pmol/L, p = 0.04).

**Conclusions:**

Calculation of serum free cortisol concentration was not helpful in identifying patients with potential hypocortisolism. SAH severity did not affect cortisol concentrations, possibly indicating relative pituitary-adrenal insufficiency in patients with more severe bleeding.

**Trial registration:**

ClinicalTrials.gov Identifier NCT00614887.

## Introduction

Recent studies suggest that disturbed glucocorticoid metabolism and adrenal insufficiency may adversely affect outcome in patients with subarachnoid haemorrhage (SAH) and traumatic brain injury (TBI) [1-5]. There is some evidence of a high incidence of delayed adrenal insufficiency (AI) in patients with SAH [5-7]. Most studies, however, have focused on TBI, and there are almost no data on the function of the hypothalamo-pituitary-adrenal gland (HPA) axis in patients with acute SAH.

In patients with SAH, the HPA axis may be affected by direct compression of the hypothalamus or pituitary gland by an aneurysm and/or blood clot. In addition, elevated intracranial pressure, vasospasm, microinfarctions of the pituitary gland, venous stasis or surgical procedures may injure these structures [8]. There is no definition of adequate HPA response in patients with SAH. For example, cut-off values for AI in patients with SAH have been derived from other patient populations, and therefore these values may not be valid in patients with SAH. Furthermore, the previous studies have focused on a single time point after SAH, and the function of the HPA axis over time has not been investigated. The relationship between serum free versus total cortisol concentrations is also not known in these patients [8].

The aim of our study was to characterise the function of the HPA axis acutely and over time for up to three months in patients with SAH. We used various methods, including serum free cortisol calculation and measurement of serum total cortisol concentration. We also performed testing of adrenocorticotropic hormone (ACTH) and measured ACTH concentrations over time.

## Materials and methods

All patients aged 18 years and older who were scheduled for elective surgical aneurysm treatment (control group) and all patients with SAH admitted to the Kuopio University Hospital in Finland between 29 March and 30 November, 2006, were prospectively assessed for eligibility for this study. The exclusion criteria for the study were any corticoid treatment (including inhaled), use of etomidate before study entry or during the study period, unknown exact bleeding day, previous history of SAH, bleeding from more than three days before inclusion, traumatic SAH, known pituitary insufficiency, and/or a moribund state of the patient. Only surgical patients were enrolled into the control group because the prescheduled hospital stay for patients with embolised aneurysms was too short. The hospital ethics committee approved the study protocol, and informed written consent was received from the patients or their next of kin.

### Patients with subarchnoid haemorrhage

The following blood samples were collected from the first to the seventh morning after bleeding: serum (s) cortisol (reference value = 170 to 540 nmol/L), s-corticoid-binding globulin (s-CBG) (reference value male = 22 to 55 μg/L; reference value female = 40 to 154 μg/L) and s-albumin (reference value = 36 to 45 g/L). Also, samples for ACTH analysis (reference value = 0 to 11 pmol/L) were collected on the first and seventh days. An ACTH-stimulating test (250 μg of tetracosactide (Synachten, Ciba-Geigy, France) administered intravenously) was performed on the first morning in the intensive care unit (ICU) and seven days after the bleeding. On the second and sixth days in the ICU, 24-hour urinary cortisol excretion (reference value = 100 to 380 nmol) was measured.

### Control patients

Blood samples were collected from control patients that were equivalent to those collected from the patients with SAH. The second ACTH sample was collected on day five. The first cortisol samples were drawn and an ACTH test was performed on day one before surgery and on day five postoperatively, and then patients were discharged. On day two, 24-hour urinary cortisol excretion was measured.

### Follow up at three months

At the scheduled three-month follow-up visit, serum total cortisol concentration was measured and free cortisol concentration was calculated at 9 am, followed by an ACTH-stimulation test performed in both groups.

Samples for serum free cortisol calculation were stored at -70°C for later analysis. The same personnel performed all analyses in one laboratory at the Kuopio University Hospital. We used electrochemiluminescence immunoassay (Elecsys Cortisol, Roche Diagnostics, Mannheim, Germany) as the diagnostic method. The Coolens method was used to calculate serum free cortisol concentration [9]: *U*^2^*K *(1 + *N*) + *U *[1 + *N *+ *K *(*G *- *T*)] - *T *= 0, where ***K***= 3 × 10^-7 ^M^**-1**^(affinity of CBG) to cortisol at 37°C, *G *= CBG, *U *= unbound cortisol, *T *= cortisol, and *N *= ratio of albumin bound to free cortisol (1.74). *U *was calculated as follows: $U=\sqrt{Z^{2}+\frac{T}{\left(1+N\right)K}}-Z$, where $Z=\frac{1}{2K}+\frac{G-T}{2\left(1+N\right)}$

Serum CBG concentrations were analysed with radioimmunoassay (BioSource Europe S.A., Nivelles, Belgium). After dichloromethane extraction, urinary free cortisol concentrations were analysed using the same method employed for serum total cortisol measurement. Plasma ACTH concentrations were analysed using an immunoluminometric assay (IMMULITE; Diagnostic Products Corporation, Los Angeles, CA).

Several serum cortisol concentration cut-off values for AI have been published. We used a random serum total cortisol concentration of less than 500 nmol/L to indicate AI [10,11]. For free cortisol, we used a concentration of less than 55 nmol/L as the cut-off for risk of AI [12]. We also tried to find out patients from our study population with low serum total (less than 350 nmol/L) and/or free cortisol concentrations (less than 22 nmol/L) and low serum ACTH concentrations (less than 5 pmol/L) to search for those at risk of secondary AI [11].

In addition, a total cortisol response less than 248 nmol/L to exogenous 250 μg ACTH [13] was used as a marker for relative AI.

### Statistical methods

We used a power of 80% and a two-sided α-level of 0.05 in sample size calculations. We assumed that 25% of patients with SAH and none of the elective surgical patients would develop AI [14], and decided to recruit 30 patients with SAH and 30 control patients.

Data are presented as mean ± standard deviation (SD) or as absolute values and percentages or medians and interquartile ranges. Distribution of the parameters was assessed by the Kolmogorov-Smirnov test. For normally distributed parameters, student's *t*-tests were used to compare the means of different groups. The Mann-Whitney U test was used for nonparametric testing between the groups. A mixed models method was used for testing between the groups, allowing heterogeneity between the groups.

Spearman or Pearson correlations were calculated. SPSS 14.0 software (SPSS, Chicago, IL) was used to perform the analyses.

## Results

**Figure 1:**
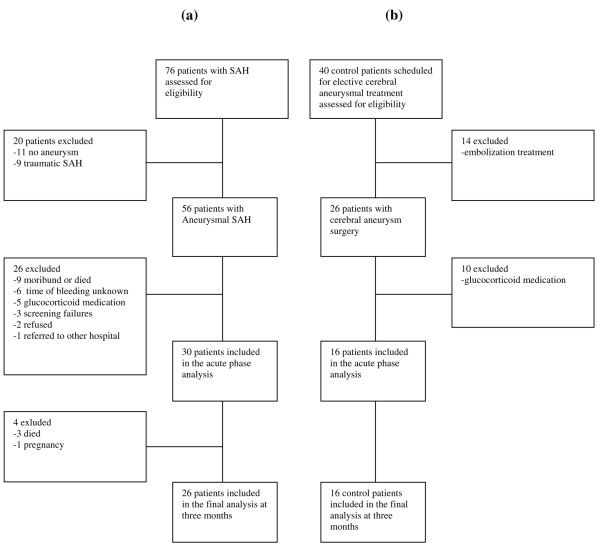
Flow chart of patients with (a) subarchnoid haemorrhage (SAH) and (b) control patients.

Thirty patients with SAH and 16 control patients assessed for elective cerebral aneurysm operations were enrolled into the study (Figures 1a and 1b). Recruitment was very slow because of the frequent choice of endovascular aneurysm treatment and high rate of exclusion for other reasons, and we were unable to enroll the planned 30 surgical patients into the control group. Seventeen patients with SAH underwent endovascular treatment and 13 patients had open surgery. The mean length of stay (LOS) for patients with SAH was 97 ± 100 hours at the ICU and 34 ± 51 hours at the high-dependency unit (HDU). Patients in the control group had an LOS in the HDU of 22 ± 2 hours. Patients with SAH had a hospital LOS of 14.6 ± 5.4 days and patients in the control group had a hospital LOS of 8 ± 3 days (p < 0.001). The three-month mortality was 10% in patients with SAH and none of the patients in the control group died. Demographic data for the patients are presented in Table 1.

**Table 1:** Demographic data of the patients

	SAH (n = 30)	Control (n = 16)	p value
Age, years (range)	52 (21 to 78)	50 (37 to 64)	0.53
Gender M/F	14/16	4/12	0.15
Aneurysm location			
ICA	6	6	
MCA	8	10	
AcoA	11	0	
ACA distal	2	0	
VBA	3	0	
Hydrocephalus at admission	6	0	
Fisher grade^16^			
I to II	4		
III to IV	26		
Hunt-Hess initial^15^			
I to II	15		
III	6		
IV to V	9		
SAPS II	30 ± 13		
APACHE II	15 ± 6		

Data are presented as numbers or mean ± standard deviation unless otherwise indicated. ACA = anterior cerebral artery; AcoA = anterior communicating artery; APACHE = Acute Physiology and Chronic Health Evaluation; HDU = high dependency unit; ICA = internal carotid artery; ICU = intensive care unit; LOS = length of stay; MCA = median cerebral artery; SAH = subarachnoid haemorrhage; SAPS = Simplified Acute Physiology Score. VBA = vertebro basilar artery.

**Figure 2:**
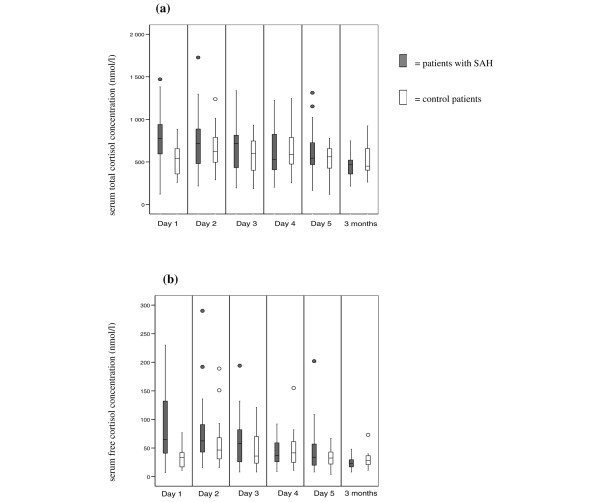
**Serum free and total cortisol concentrations**. Patients with subarchnoid haemorrhage (SAH) had higher initial (a) serum total cortisol (p = 0.001) and (b) free cortisol concentrations (p < 0.001) than control patients. At later time points, there were no differences between the groups in this respect. Data are presented as median, interquartile ranges and outliers.

Serum total and free cortisol concentrations are presented in Figure 2. In the control patients, the mean random serum total cortisol concentrations were 535 ± 193 nmol/L at day one, 666 ± 244 nmol/L at day two, 571 ± 224 nmol/L at day three, 641 ± 261 nmol/L at day four, 534 ± 178 nmol/L at day five and 517 ± 185 nmol/L at three months. The mean random serum free cortisol concentrations were 33 ± 18 nmol/L at day one, 61 ± 48 nmol/l at day two, 46 ± 33 nmol/L at day three, 49 ± 36 nmol/L at day four, 34 ± 18 nmol/L at day five and 31 ± 17 nmol/l at three months.

In the patients with SAH, the mean random serum total cortisol concentrations were 793 ± 312 nmol/L at day one, 736 ± 320 nmol/L at day two, 679 ± 271 nmol/L at day three, 620 ± 258 nmol/L at day four, 596± 262 nmol/L at day five and 454 ± 124 nmol/L at three months. The mean random serum free cortisol concentrations were 83 ± 55 nmol/L at day one, 74 ± 57 nmol/L at day two, 63 ± 44 nmol/L at day three, 44 ± 24 nmol/L at day four, 46 ± 40 nmol/l at day five and 24 ± 9 nmol/L at three months.

**Figure 3:**
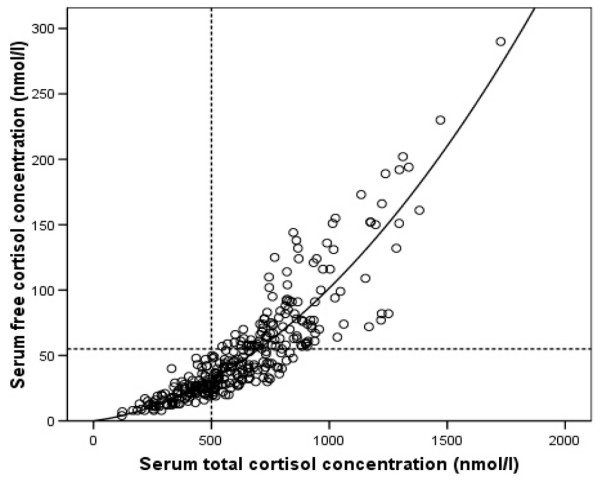
**Pooled data and quadratic regression analysis**. Equation from quadratic regression analysis for serum free cortisol concentration = 0.024 × serum total cortisol concentration + 0.0000772 × total cortisol concentration^2^. Pearson correlation coefficient 0.88, p < 0.001.

The dependence of free cortisol on total cortisol (Figure 3) shows the expected hyperbolic increase of free cortisol with total cortisol, until CBG is saturated, when the relation becomes linear. There was a good correlation between serum total and free cortisol concentration at day one (r = 0.66, p < 0.001), day three (r = 0.9, p < 0.001), day five (r = 0.886, p < 0.001) and three months (r = 0.75, p < 0.001) (Figure 3).

**Figure 4:**
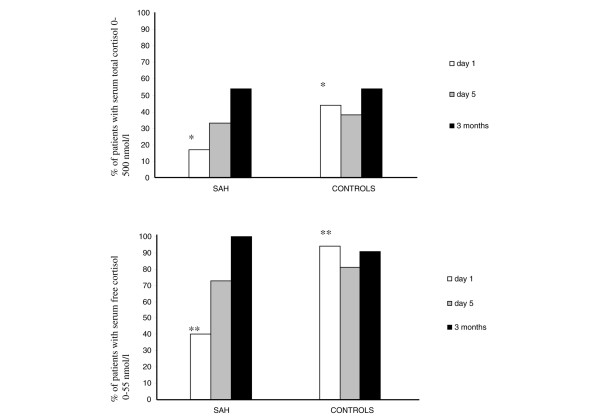
**Percentage of patients with baseline serum free cortisol concentration less than 55 nmol/L or serum total cortisol concentration less than 500 nmol/L**. * = difference between the groups at day one, p = 0.046. ** = difference between the groups at day one, p < 0.001.

The percentages of patients whose serum total cortisol concentration was less than 500 nmol/L or with a serum free cortisol concentration less than 55 nmol/L are presented in Figure 4.

There was a significant difference between control patients and patients with SAH at day one in serum total (p = 0.001) and free (p = 0.001) cortisol concentrations. There was no difference in serum total or free cortisol concentrations through the study period in patients with SAH divided into groups with GCS at the first 24 hours of less than eight (n = 9) or more than eight. The cortisol concentrations also did not differ between patients who required (n = 9) or did not require noradrenaline due to symptomatic vasospasm and/or cerebral perfusion pressure maintenance. Hydrocephalus at admission (n = 6) or hypoalbuminea (< 25 g/L) (n = 8) did not affect serum free or total cortisol concentrations in patients with SAH. In addition, patients with SAH and a serum albumin of either less than 25 g/L or more than 25 g/L had equal free/total cortisol concentration ratios. Bleeding severity according to Hunt-Hess grade [15] did not affect serum free or total cortisol concentrations. There was neither any difference in the serum free or total cortisol concentrations if the patients had hyponatraemia (p-natrium < 130 mmol/L) at any time during the study. No patient had sepsis. Control patients received cloxacillin twice at the first day. Patients with SAH received cloxacillin for prophylaxis if they had intraventricular pressure monitoring.

At day one, patients with SAH had an ACTH concentration of 5.3 ± 4.7 pmol/L; in the control patients, the concentration was 4.4 ± 2.2 pmol//L (p = 0.88). At day seven, the ACTH concentration was 5.6 ± 4.2 pmol/L in patients with SAH and 4.4 ± 1.3 pmol/L at day five in the control group (p = 0.43). Serum ACTH correlated with serum free cortisol concentration at day one (r = 0.32, p = 0.045). Otherwise serum total or free cortisol concentrations did not correlate with serum ACTH concentration. There were two patients with SAH who had low serum total cortisol concentrations (122 nmol/L and 303 nmol/L) and low serum free cortisol concentrations (7 nmol/L and 21 nmol/L) combined with low ACTH concentrations (3.08 pmol/L and 2.2 pmol/L) at the first day: they both had normal values afterwards. The other patient of these two also failed the three-month ACTH test (ACTH-response < 248 nmol/l).

After one week there were two patients with low serum total cortisol concentrations (254 nmol/L and 335 nmol/L) and low serum free cortisol concentrations (14 nmol/L and 14 nmol/L) with a combination of low serum ACTH concentrations (2.64 pmol/L and 2.2 pmol/L). Additionally there were two patients with isolated low serum free cortisol concentrations (20 nmol/L and 21 nmol/L) and normal total cortisol concentrations (573 nmol/L and 474 nmol/L) but low ACTH concentrations (4.4 pmol/L and 3.5 pmol/L).

Treatment modality or aneurysm location (anterior communicating artery versus other artery) did not affect serum ACTH or free or total cortisol concentrations. Patients with Hunt-Hess grades IV to V had higher ACTH concentrations at days one (10.7 ± 7.1 pmol/L) and five (8.2 ± 7.7 pmol/L) than patients with Hunt-Hess grades I to III (day one:3.8 ± 2.0 pmol/L, p = 0.002; day five: 4.7 ± 1.8 pmol/L, p = 0.04). GCS (< 8 or > 8), Fisher grade [16] (I-II versus III-IV), or presence of hydrocephalus did not affect ACTH concentrations.

**Figure 5:**
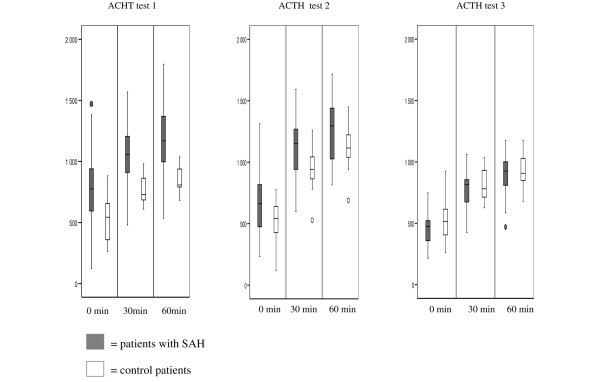
**Response to exogenous adrenocorticotropic hormone (ACTH) in patients with subarachnoid haemorrhage (SAH) and control patients**. There were no statistically significant differences between the groups. The percentage of patients with a serum cortisol response less than 248 nmol/L also was not different between the groups. Test one = day one; test two = day five (control) and day seven (SAH); test three = three months. Data are presented as median, interquartile ranges and outliers.

**Figure 6:**
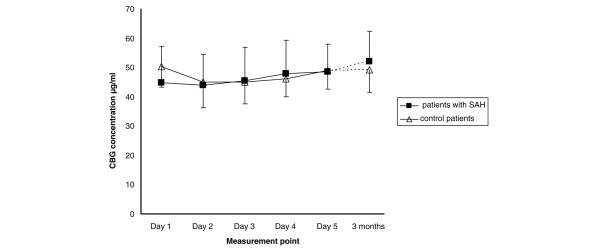
**Evolution of corticoid-binding globulin (CBG) concentration in patients with subarachnoid haemorrhage (SAH) and control patients**. There were no between-group differences, but CBG concentration changed significantly over time in both groups, p = 0.008. Data are presented as mean ± standard deviation.

Figure 5 illustrates the response to exogenous ACTH (ACTH stimulation test). At day one, eight (33%) patients with SAH and eight (50%) control patients had a cortisol response less than 248 nmol/L (p = 0.11). At day five and at three months, one patient with SAH and no control patients had a cortisol response less than 248 nmol/L (neither was significant). The patient with a failed synachten test at three months also failed the first and second test. The 24-hour urinary free cortisol concentration was 4896 ± 5342 nmol/L in patients with SAH and 1641 ± 1601 nmol/L in the control group at day two (p = 0.001). At day six, the urinary 24-hour free cortisol concentration was 3276 ± 3710 nmol/L in patients with SAH. The evolution of CBG concentration is presented in Figure 6.

## Discussion

According to our study, SAH causes an HPA axis response similar to that of elective cranial aneurysm surgery. The severity of SAH does not affect serum free or total cortisol concentrations, which could potentially reflect relative AI in patients with more severe SAH. We also found a good correlation between serum free and total cortisol concentrations; therefore calculation of serum free cortisol may not offer any advantage in the clinical setting.

One of the major stress responses to trauma is the activation of the HPA axis to increase cortisol production. SAH may disturb the function of the HPA axis by brain swelling, microinfarctions, venous stasis, surgical manipulation or hypoperfusion of the brain [8]. Some data suggest delayed insufficiency of the HPA axis after SAH [5,17,18]. Little is known of the cortisol-secretion dynamics in the very acute phase in SAH or about the function of the whole HPA axis over time in patients with SAH. Studies in acute neurosurgical patients include only a very few patients with SAH and predominantly patients with TBI [19,20]. Additionally, some patients in previous studies had received glucocorticoid treatment [21]or only a single time point was investigated [19]. However, we could not demonstrate any difference in the behaviour of serum total cortisol concentrations between patients with SAH and patients in the control group. Furthermore, although open aneurysm surgery was supposed to cause a clearer elevation of serum cortisol concentrations than endovascular treatment, we did not observe any difference in the HPA function between patients with surgical and endovascular treatment of their ruptured aneurysms. Neither were we able to show any difference in the adrenal response between comatose (GCS < 8) and noncomatose patients. This indicates that adrenal response may be inadequate, that is it may be insufficient in the comatose and therefore more severely ill patients.

We are not aware of other studies that provide calculation of serum free cortisol concentration in patients with SAH. Cortisol is bound about 90% to CBG and albumin, and only the unbound free fraction is responsible for the physiological effects. By measuring CBG, it is possible to obtain the serum free cortisol concentration using the Coolens' method [9] which has been shown to be reliable in ICU patients [22]. CBG has a low capacity and high affinity for cortisol and it becomes saturated at a serum cortisol concentration of about 690 nmol/L [11]. This is seen in Figure 3 as a change in the slope of the correlation lines. Any alterations in plasma albumin and CBG concentration may affect the relationship between serum free and total cortisol concentrations; however, this is not seen in Figure 3 as the majority of the data follow a similar trend. This is to be expected as CBG concentrations are not grossly affected in SAH (Figure 6).

It has also been suggested that serum albumin may affect testing of adrenal function in critically ill patients [12]. We found that calculation of serum free cortisol concentration was not more informative than measurement of serum total cortisol concentrations. Furthermore, we did not see any difference in serum free or total cortisol concentrations in hypoalbumenic versus other patients. Neither did we observe any difference in CBG concentration in hypoalbumenic patients as compared with patients with an albumin concentration more than 25 g/L. This finding is in accordance with those of a previous study by Ho and colleagues in which they found that adjustment of the Coolens' equation constant to albumin concentration had only a minimal effect on estimated serum free cortisol [22].

ACTH concentrations were higher in patients with Hunt-Hess grades IV to V compared with patients with Hunt-Hess grades I to III. It remains unclear if the ACTH response is sufficient or if the adrenal gland responds adequately to ACTH. The adrenal gland response is likely to be unaffected because the cortisol response to exogenous ACTH was adequate. It is possible that SAH already causes ACTH deficiency and secondary AI in the acute phase of bleeding and some studies suggest that this may last years after the primary ictus present [5,17]. As an indication of this, we found patients with SAH who had low serum free or total cortisol concentrations and low ACTH concentrations reflecting possible secondary AI. We found no correlation between serum ACTH concentration and serum free or total cortisol concentrations. This finding is not in accordance with that of Tanriverdi and colleagues [23] who identified a correlation between serum cortisol and ACTH concentration with measures at a single time point only. It must be remembered that in stress situations there is also non-ACTH-mediated cortisol secretion caused by inflammatory cytokines for example [24]. Medication of the patients may affect cortisol secretion as well [3]. Patients in the present study did not receive any etomidate.

Critically ill patients who have relative adrenal insufficiency confirmed by the ACTH-stimulation test may benefit from cortisol-substitution therapy [25]. We observed similar incidences of adrenal insufficiency in both the patients with SAH and the control group when the ACTH-stimulation test was performed repeatedly. In critically ill patients, the value of ACTH testing has recently been questioned [26], especially the value of this test in detecting AI at high baseline cortisol concentration which might tell us of an exhausted adrenal gland rather than real adrenal insufficiency. This test may not be reliable for detecting adrenal insufficiency in critically ill patients, and confounding factors influence the method by which cortisol is analysed [27,28]. Despite our efforts, it remains unclear whether the HPA axis response in patients with SAH is adequate.

There are several different classifications and diagnostic criteria for detecting AI and they differ in sensitivity and specificity [29]. These criteria are based either on random serum total cortisol concentration [10,30] or on the ACTH-stimulation test [13]. Depending on the criteria used, the incidence of AI varies widely, as it does, for example, in patients with TBI [4]. It is noteworthy, however, that the diagnostic criteria have been derived from studies on patients who do not have acute intracranial pathology. Specifically, there are no studies that focus on the acute phase of SAH. Hence, the definitions for HPA axis deficiency are not available for these patients. In our study, the incidence of adrenal insufficiency in SAH varied between 17% and 50% depending on the different existing definitions used for critically ill patients in general. In this respect, we did not find any difference in AI between patients with SAH or control patients. Despite recent suggestions, our findings indicate that it is premature to recommend glucocorticoid treatment for patients with SAH [8]. For example, we found much higher 24-hour urinary free cortisol concentrations in patients with SAH compared with control patients, which could support an assumption of the presence of adequate cortisol dynamics.

There are some limitations in our study. Although we used versatile methods to investigate the HPA axis response over time, we could not evaluate the period between one week and three months. To choose another control group, for example septic or trauma patients, would have caused numerous additional confounding factors including corticoid therapy, infections and repeated operations. We were also unable to recruit as many patients into the control group as we originally planned. Our study was not powered for subgroup analysis. In addition, the number of patients with severe bleeding was limited, which could potentially have affected our results.

## Conclusions

For the first time the function of the HPA axis in patients with acute SAH was characterised over time, up to three months, using various methods. Our findings suggest that SAH causes a rapid cortisol response, but whether this response is adequate in more severely ill patients remains unclear. The clinical picture of our patients does not support AI in patients with SAH. Calculation of serum free cortisol concentrations does not seem to be helpful in assessing adrenal function in patients with SAH.

## Key messages

• This is the first study to investigate adrenal function in patients with clearly defined acute aneurysmal SAH

• Severe SAH causes various rises in cortisol and ACTH; some patients have values that could be inappropriate for the amount of stress sustained

• Calculation of serum free cortisol is not helpful in assessing adrenal function in SAH

• More studies are needed to define normal HPA response in acute SAH

## Abbreviations

ACTH: adrenocorticotropic hormone; AI: adrenal insufficiency; CBG: corticosteroid binding globulin; GCS: Glascow Coma Scale; HDU: high-dependency unit; HPA: hypothalamo-pituitary-adrenal; ICU: intensive care unit; LOS: length of stay; SAH: subarachnoid haemorrhage; SD: standard deviation; TBI: traumatic brain injury.

## Competing interests

This study was supported in part, by academic grants from Maire Taponen Foundation, Helsinki, Finland and Kuopio University Hospital Grant, Kuopio, Finland. The authors declare that they have no competing interests.

## Authors' contributions

SB, AU and TK participated in study conception, acquisition of data, analysis and interpretation of data, drafting the manuscript and critically revising it. ER participated in study conception, analysis and interpretation of data, drafting the manuscript and critically revising it. JRI and IV participated in study conception, manuscript drafting and critically revising it. JRO participated in analysis and interpretation of data, drafting the manuscript and critically revising it. VK participated in analysis and interpretation of data and in revising the critically manuscript.
